# Comparative impact of nanoparticles on salt resistance of wheat plants

**DOI:** 10.1016/j.mex.2023.102371

**Published:** 2023-09-09

**Authors:** Adeoke Olatunbosun, Huseynova Nigar, Khalilov Rovshan, Amrahov Nurlan, Jafarzadeh Boyukhanim, Abdullayeva Narmina, Azizov Ibrahim

**Affiliations:** a Baku State University, Baku, Azerbaijan; b Ministry of Agriculture, Azerbaijan Research Institute of Crop Husbandry, Baku, Azerbaijan; c Institute of Molecular Biology and Biotechnologies, Baku, Azerbaijan

**Keywords:** Salt tolerance, Fe_3_O_4_, ZnO, Al_2_O_3_, CuO NPs, *Triticum aestivum* L, Treatment of crops with nanoparticles

## Abstract

When it comes to climate change, salt stress is a significant danger to agriculture and can lead to decreased crop yields due to various factors such as osmotic and ionic stress, as well as oxidative stress, disruption of hormone balance, and nutrient imbalance (Fig. 2). Despite this, there is a growing pressure to expand agriculture into salt-affected regions to meet the demands of a growing population.

• Research has shown that supplementing plants with nanoparticles can help them adapt and alleviate the negative effects of salt stress.
• Different types of nanoparticles and nanofertilizers have shown potential in managing salt stress. This review focuses on recent progress in using Fe_3_O_4_, ZnO, Al_2_O_3_ and CuO nanoparticles to improve salt tolerance in wheat plants and highlights future research directions in this area.
• The study utilized nanoparticles to investigate their impact on plant morphology and photosynthesis intensity, including chlorophyll and carotenoid content, as well as light spectrum absorption in common wheat (*Triticum aestivum L.*).

## Graphical abstract

**Figure:**
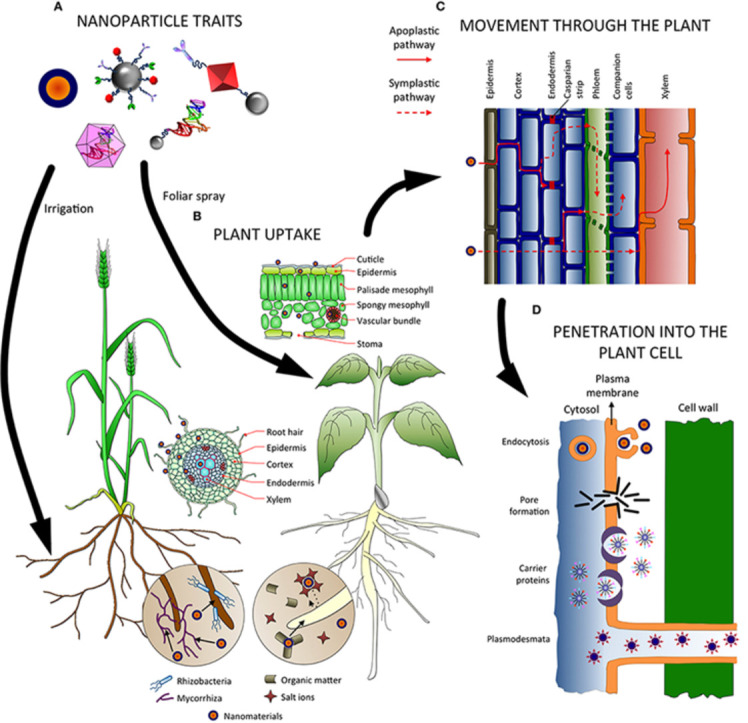

Specifications table

**Table:** 

Subject area:	Agricultural and Biological Sciences
More specific subject area:	Crop husbandry
Name of your method:	Treatment of crops with nanoparticles
Name and reference of original method:	M. Faizan, S. Hayat, and J. Pichtel, “Effects of zinc oxide nanoparticles on crop plants: a perspective analysis,” Sustainable Agriculture Reviews, Springer Link (41), (2020), pp.83–99 doi: https://doi.org/10.1007/978–3-030–33996–8_4
Resource availability:	*Equipment:* Spectrophotometry Vortex mixer Laboratory scales *Objects:* *Seed of wheat* (*Triticum aestivum L*) variety of “Qobustan”
	*Reagents:* Fe_3_O_4_, ZnO, Al_2_O_3_ and CuO nanoparticles NaCl Coco coir (compressed bricks) Ca (NO_3_)_2_ · 4H_2_0 KNO_3_ MgSO_4_ · 7H_2_0 KH_2_PO_4_ H_3_BO_3_ MnSO_4_ · H_2_0 ZnSO_4_ · 7H_2_0 Na_2_MoO_4_ · 2H_2_0 CuSO_4_ Titriplex III (EDTA – C_10_H_14_O_8_N_2_Na_2_ · 2H_2_O) FeSO_4_ · 7H_2_O H_2_SO_4_ Acetone

## Introduction

**Fig. 1:**
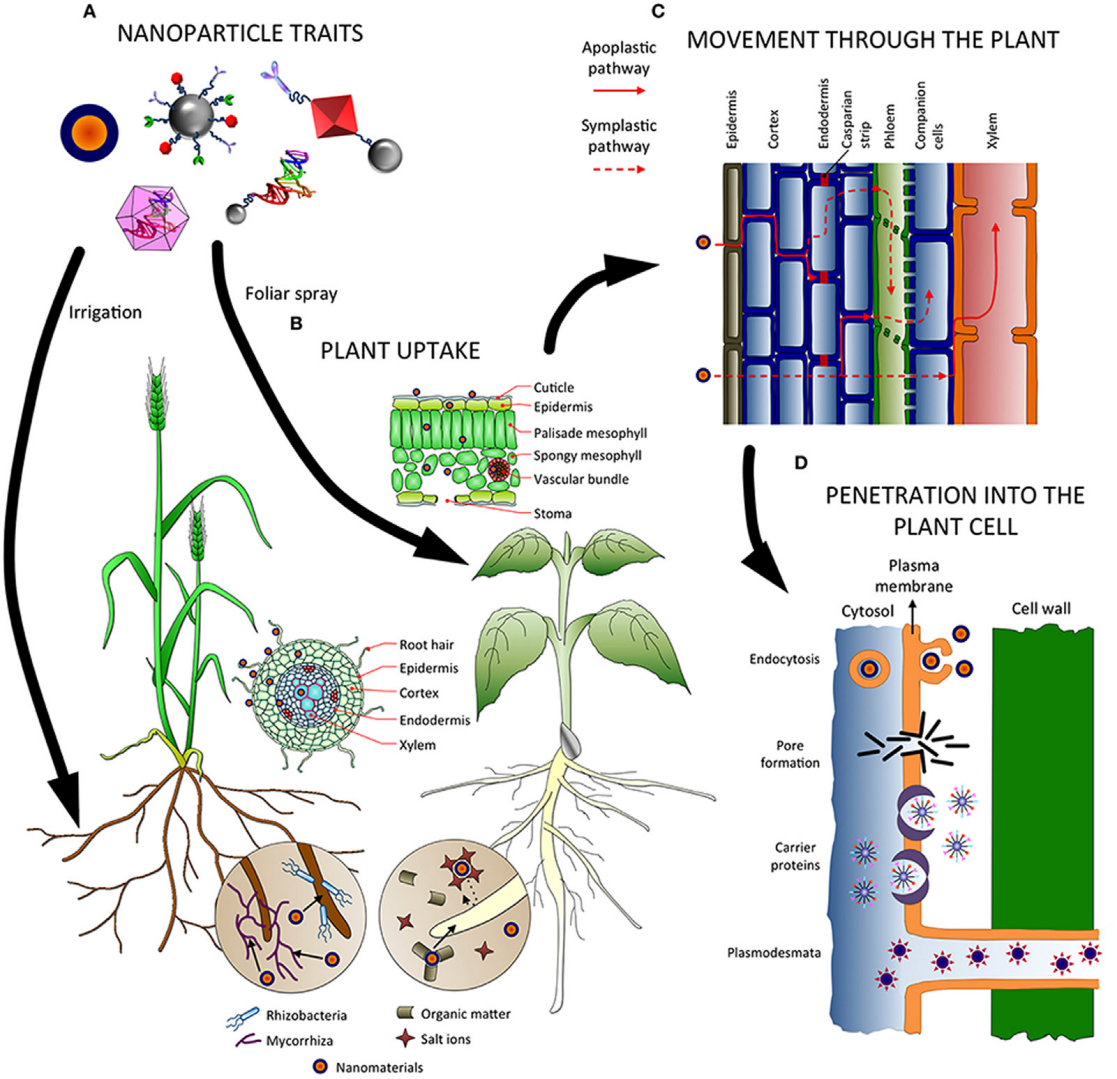
Factors affecting the absorption, uptake, transportation, and penetration of nanoparticles within plants. (A) NP traits; (B) uptake of NPs from the soil; (C) movement of NPs through the plant; (D) uptake of NPs within cellular structures.

Nanoparticles (NPs) are very small and persistent materials that are insolouble and range in size from 1 to 100 nm [1,2]. They exist in various shapes and concentrations in the environment [3,4] and research on NPs has increased in the 20th century [5]. They can be classified into categories like organic, inorganic, natural, and artificially synthesized [6]. Nanoparticles, such as Fe_3_O_4_, ZnO, and SiO2, are currently under research and have demonstrated a positive effect on the germination process of plants. They achieve this by reducing the adsorption of toxic elements through nanoparticles and by effectively combating plant pathogen. They hold the potential to serve as promising candidates for targeted drug delivery in plants [7], [8], [9], [10]. Copper (Cu) in low concentration is widely recognized as an essential micronutrient for plants and animals, particularly at low concentrations. Moreover, apart from its function as a micronutrient, Cu is also utilized to manage fungal and bacterial diseases in various crop plants [11]. Nanoparticles enter the environment through various pathways [12] and can penetrate plants through their roots, leaves or stems illustrated in Fig. 1 [13]. They travel through the vascular tissues of the plants or the free intercellular space in the ascending direction [14]. Research on the impact of NPs on plant physiology, specifically photosynthesis, which produces oxygen and edible carbohydrates, is relevant. These impacts could include alterations in the rate of photosynthesis, changes in the production of oxygen and edible carbohydrates, and potentially any associated physiological responses or modifications in the plants' growth and development. [15,16]. The size, concentration, and starting material of NPs determine their positive or negative effects on plant growth and development [17]. Low or medium concentrations of NPs can promote plant growth, increase oxygen production, and intensify CO_2_ absorption [18]. However, high concentrations of NPs can be cytotoxic and genotoxic and damage various cells [19]. ZnO can damage the tonoplast surrounding the vacuole, leading to decreased photosynthetic intensity and water conductivity [12]. Fe_3_O_4_ NPs can reduce the weight of roots and leaves, and decrease the content of carotenoids and chlorophyll a and b in leaves, promoting chloroplast cell degradation [20]. Soil salinity poses a significant threat to agriculture, as it can negatively impact crop productivity by inducing osmotic stress and ion toxicity. The ions that are primarily responsible for this are sodium, chloride, calcium, magnesium, sulfate, potassium, bicarbonate, carbonate, nitrate, and occasionally borate ions [21,22].

**Fig. 2:**
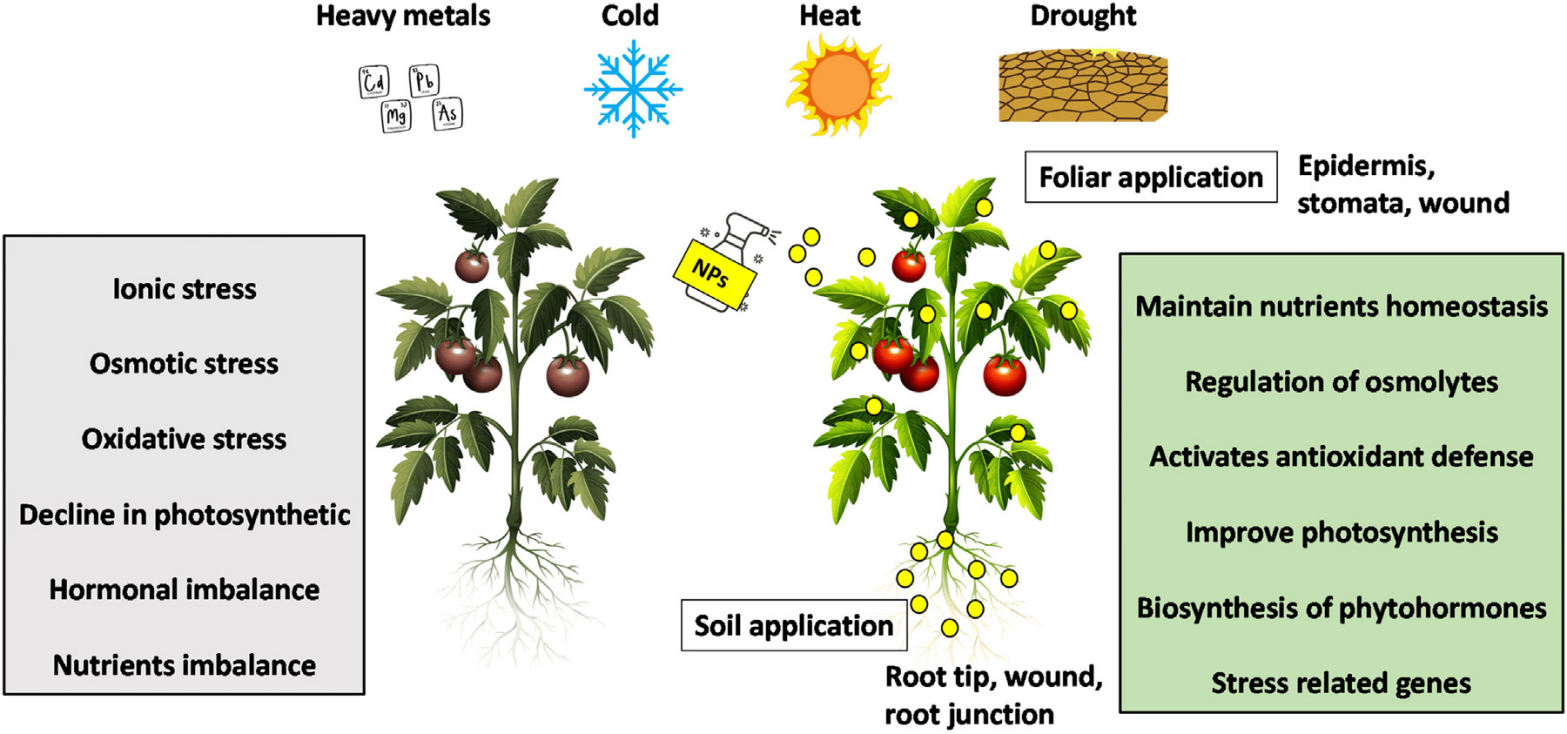
NP mechanisms for alleviating abiotic stresses in plants.

## Method details

The specific experiment being reffered was undertaken in the Biophysics and biochemistry Research laboratory at Baku State University, Baku, Azerbaijan. Seeds of wheat (*Triticum aestivum L*) variety of “Qobustan” were obtained from the Institute of Botany and stored at 10°C in moisture-proof polyethylene bags after receiving, until the start of experiments during 10 days. NPs (Fe_3_O_4_, ZnO, Al_2_O_3_ and CuO) were obtained from the State Oil Company of Azerbaijan (SOCAR).

### Treatment seeds with nanoparticles

Dry powder seed treatment (DS) method is employed for subjecting seeds to nanoparticles. This approach is favored to prevent nanoparticles from aggregating or precipitating in water. For DS treatment, a total of thirty (30) wheat seeds were utilized. First, mechanical scarification was done by sandpaper. The surface of the seeds was gently obstructed between the sheets of sandpaper to scratch the seed surface. After scarification, seeds were dressed in nanoparticles by mixing seeds with nanoparticle powder. 50 mg of each nanoparticle (Fe_3_O_4_, ZnO, Al_2_O_3_ and CuO) was mixed with the 30 selected seeds (in total 1.4 gr) in glass vials.

### Common wheat seedling cultivation

*Wheat soil preparation*: (a) For five Petri dishes, 40 gs of coco coir weighed by an electronic balance was mixed with 0.24 gs of crystalline NaCl and wet with 15 ml of water. This resulted in 0.6% salinity level of growing medium.(b) For five pots, 500 gs of coco coir weighed by an electronic balance was mixed with 3 gs of crystalline NaCl and wet with 15 ml of water. This resulted in 0.6% salinity level of growing medium. In total, ten pots (groups) were prepared. Each had 10 wheat seedlings. Seedlings in two groups were labelled “control” and “salt-stressed”; the eight left were “treated”. Control was neither treated with nanoparticle nor salt. Salt stressed was not treated with nanoparticle but sown in salted condition. Four of the remaining eight were treated with a nanoparticle and sown in unsalted condition while the other four were treated with a nanoparticle but sown in salted condition.

*Preparation of hydrophone solution:* The Steiner nutrient solution was prepared by adding 10 ml of solution A and B and 1 ml of solution C and D to 1 L of tap water. The solution was then aerated for 16 hours. Afterwards the pH was measured and adjusted at pH 6.5±0.5 using no more than 158ul concentrated sulphuric acid per litre solution. Solution A is 68 g Ca (NO_3_)_2_ · 4H_2_0 + 62 g KNO_3_ per litre water. Solution B is 46 g MgSO_4_ · 7H_2_0 + 13.6 g KH_2_PO_4_ per 1 L water. Solution C is 2.69 g H_3_BO_3_ + 2.00 g MnSO_4_ · H_2_0 + 0.506 g ZnSO_4_ · 7H_2_0 + 0.126 g Na_2_MoO_4_ · 2H_2_0 + 0.078 g CuSO_4_ · 5H_2_0 per 1 L water. Each chemical was dissolved separately in 100 ml of tap water, where H_3_BO_3_ is dissolved (±70 °C). Solution D1 is 16.659 g Titriplex III (EDTA – C_10_H_14_O_8_N_2_Na_2_ · 2H_2_O) + 2.91KOH per 500 ml distilled water. The titriplex was dissolved in hot distilled water (±70 °C). Solution D2 is 12.44 g FeSO_4_ · 7H_2_O + 2 ml 0.5 M H_2_SO_4_ per 200 ml distilled water. Solution D is obtained when D1 and D2 were added and supplemented until 900 ml, aerated and supplemented until 1 litre with distilled water. A reddish brown coloured solution was seen. The solutions A, B, C and D were joined, supplemented again until 1 L.

The wheat seeds were used for experiment were soft spring-crop wheat, purchased from the Institute of Botany in Baku, Azerbaijan. To eliminate the influence of other nutrients on seedling growth, the seeds were germinated on Petri dishes soaked in water only and transferred to a growth chamber and grown hydroponically in all experimental groups for 15 days at a temperature of +24 °C. The water was regularly added to ensure consistent moisture levels, and the seeds were observed daily. Plants were treated with 0% (control) and 0.6% sodium chloride (NaCl). Coco coir (compressed bricks) was the growing medium used for the experiments. As moderate salinity serves as a stressor, the electrical conductivity (EC) level in the nutrient medium should be kept to a minimum. The EC level in the nutrient medium should be maintained at a low level. This is to ensure that the concentration of salts in the medium is controlled and not too high, which could have adverse effects on the plants' response to the stressor. Due to its very low EC and almost zero charge, coco coir allows for complete control over the nutrients fed to plants. Weights of coco coir were measured and placed in petri dishes and pots. The coco coir was manually mixed with NaCl.

**Fig. 3:**
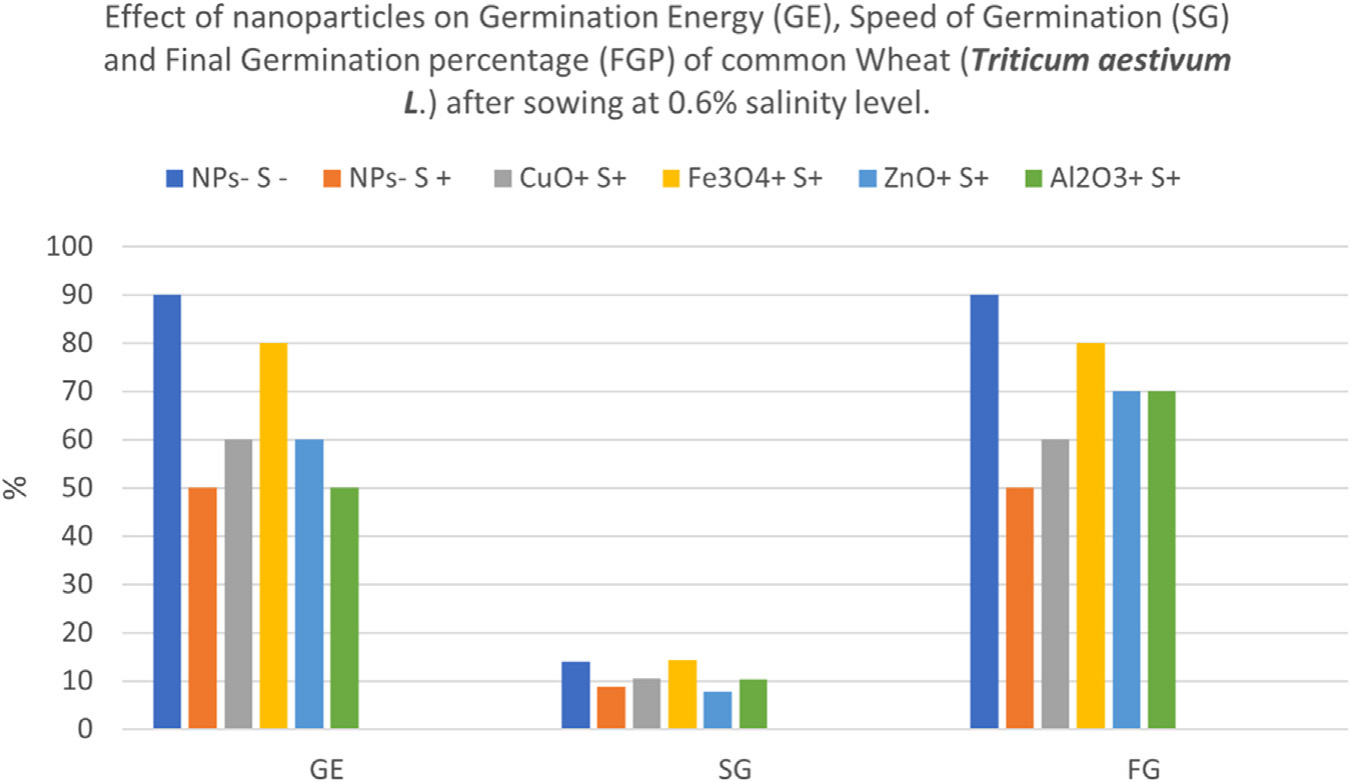
NPs- S- (not treated with nanoparticle nor salted), NPs- S+ (not treated with nanoparticle but salted), NPs+ S- (treated with nanoparticle but not salted), NPs+ S+ (treated with nanoparticle and salted).

**Fig. 4:**
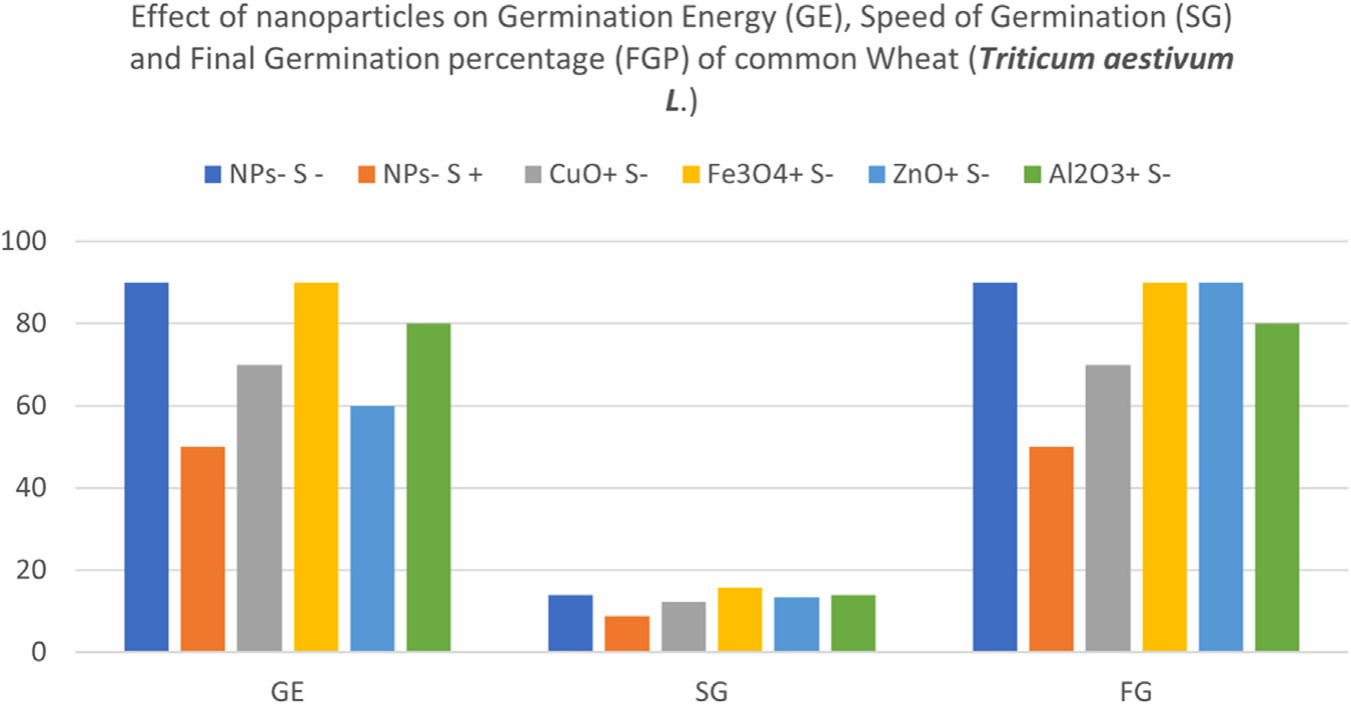
NPs- S- (not treated with nanoparticle nor salted), NPs- S+ (not treated with nanoparticle but salted), NPs+ S- (treated with nanoparticle but not salted), NPs+ S+ (treated with nanoparticle and salted).

Effects of CuO, Fe_3_O_4_, ZnO and Al_2_O_3_ nanoparticles on Germination Energy (GE), Speed of Germination (SG) and Final Germination percentage (FGP) of common Wheat (*Triticum aestivum* L.) after sowing in both 0.6% saline and Non-saline conditions is shown in Figs. 3 and 4. At moderately saline conditions of 0.6%, salt-stressed seeds (NPs- S+) and seeds treated with Al_2_O_3_ NPs had lowest germination energy of 50% each, while CuO and ZnO fared a bit better having values of 60% each. Of all treated seeds, Fe_3_O_4_ NPs showed highest salt tolerance with GE value of 80% though 10% less than control (NPs- S-) with 90%. When speed of germination was examined, Fe_3_O_4_ NPs treated seeds were the quickest, followed by control seeds, CuO, Al_2_O_3_, salt-stressed seeds and finally, ZnO NPs seeds as the least.

In non-saline conditions shown in Fig. 4, salt-stressed seeds had the lowest GE value of 50%, while control seeds had the highest with 90%. Seeds treated with ZnO, CuO and Al_2_O_3_ NPs demonstrated higher salt tolerance than salt-stressed seeds with GE values of 60%, 70% & 80% respectively. Of all treated seeds, those with Fe_3_O_4_ NPs equalled the GE value of control. Seeds treated with Fe_3_O_4_ NPs germinated quickest while salt-stressed seeds were the slowest.

### Morphological analysis of common wheat seedlings

The experiment involved evaluating the morphological parameters of seedlings from all groups. Samples selected from each group were healthy and did not have any lesions or pathogens. The seedlings were grown hydroponically treated with and without NPs for 15 days, during which the length of the leaves of the shoots were measured using a ruler. Additionally, the number of root shoots was also counted.

**Fig. 5:**
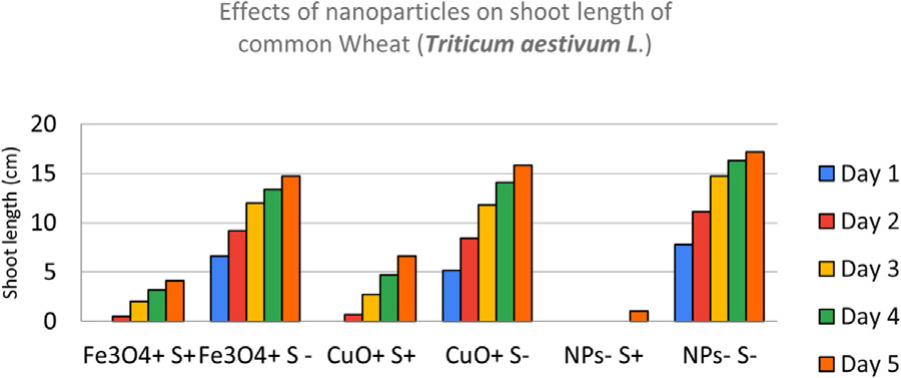
NPs- S- (not treated with nanoparticle nor salted), NPs- S+ (not treated with nanoparticle but salted), NPs+ S- (treated with nanoparticle but not salted), NPs+ S+ (treated with nanoparticle and salted).

**Fig. 6:**
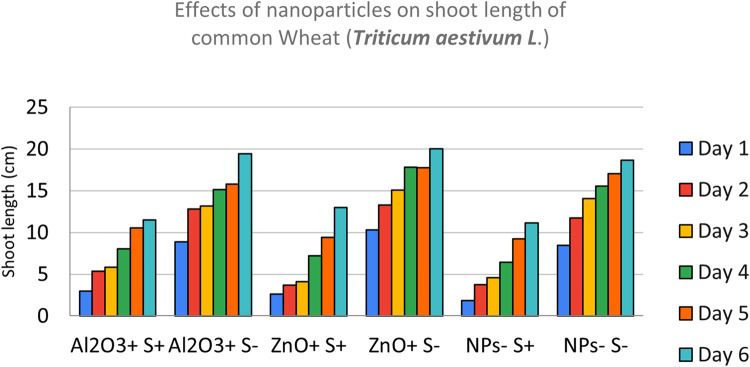
NPs- S- (not treated with nanoparticle nor salted), NPs- S+ (not treated with nanoparticle but salted), NPs+ S- (treated with nanoparticle but not salted), NPs+ S+ (treated with nanoparticle and salted).

Effects of CuO, Fe_3_O_4_, ZnO and Al_2_O_3_ nanoparticles *on shoot length of common Wheat (*Triticum aestivum L.) after sowing in both 0.6% saline and Non-saline conditions was shown in Figs. 5 and 6. The shoot of wheat seedlings across various nanoparticles combinations were measured using a ruler over a period of 5 to 6 days. At moderately saline conditions of 0.6%, salt ions induced physiological stress that limits cell division and elongation of shoots. Salt stressed seeds barely recorded any growth, those treated with Fe_3_O_4_ NPs fared better while CuO NPs treated seeds fared best. Obviously, there is an overall negative effect of salt on shoot length across all NP combinations.

### Preparation of plant suspension for photosynthetic pigments extraction

The wheat seedlings were cut into small pieces and 100 mg of green leaves from common wheat were weighed for each seedling. To the weighed leaves, 5 ml of 96.6% acetone was added and the mixture was ground with a pestle. 20 ml acetone was further added to the extract to make a total of 25 ml. The resulting mixture was then filtered using a new filter for each sample. The mixture was kept in the dark for 30 minutes before measurements.

### Determination of Photosynthesis Intensity by Spectrophotometry

The absorbence of photosynthetic pigments (chlorophyll a, chlorophyll *b* and carotenoids) in the mixture was determined using a UV/Vis spectrophotometer. Measurements were carried out on blank and true samples. The obtained data were collected at light wavelengths of 662, 644 and 440 nm for chlorophylls (a and b), and carotenoids respectively.

(A) The pigment concentrations were calculated using Von Wettstein's formula as following:
  Chlorophyll a = 9.784 × A662 – 0.99 × A644
  Chlorophyll *b* = 21.426 × A644 – 4.65 × A662;
  Carotenoids = 4.695 × A440 – 0.268 × (Chl a + b).

The concentration of pigments was expressed in mg/g of fresh weight of leaves according to the following formula:

mg/g = (mg/l × dilution) / (sample weight W × 1000)

where A is the absorption in a spectrophotometer at a specifc wavelength (nm); W is the mass of the fresh sample (g).

(B) Germination Energy (GE), Speed of Germination (SG) and Final Germination percentage (FGP) were calculated using the following formulas [23]
  GE (%) = (Number of germinated seeds at 4 DAS/Total number of seed tested) × 100
  SG = Number of germinated seeds/Days of first count + ………….. + Number of germinated seeds/Days of final count (9 days)
  FGP = (Number of total germinated seeds/Total number of seed tested) × 100

**Fig. 7:**
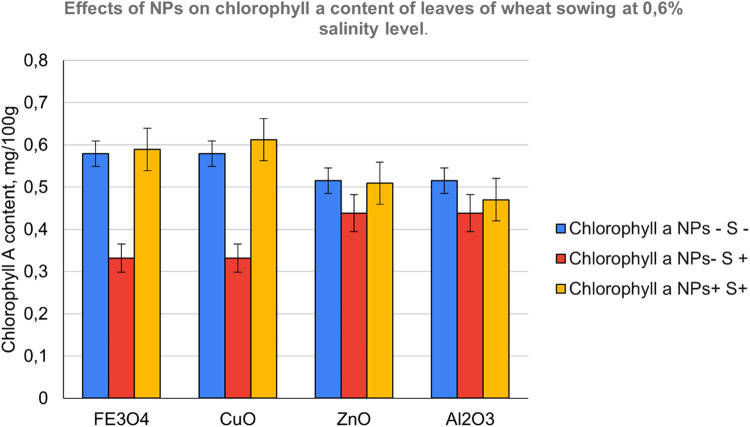
NPs- S- (not treated with nanoparticle nor salted), NPs- S+ (not treated with nanoparticle but salted), NPs+ S- (treated with nanoparticle but not salted), NPs+ S+ (treated with nanoparticle and salted).

**Fig. 8:**
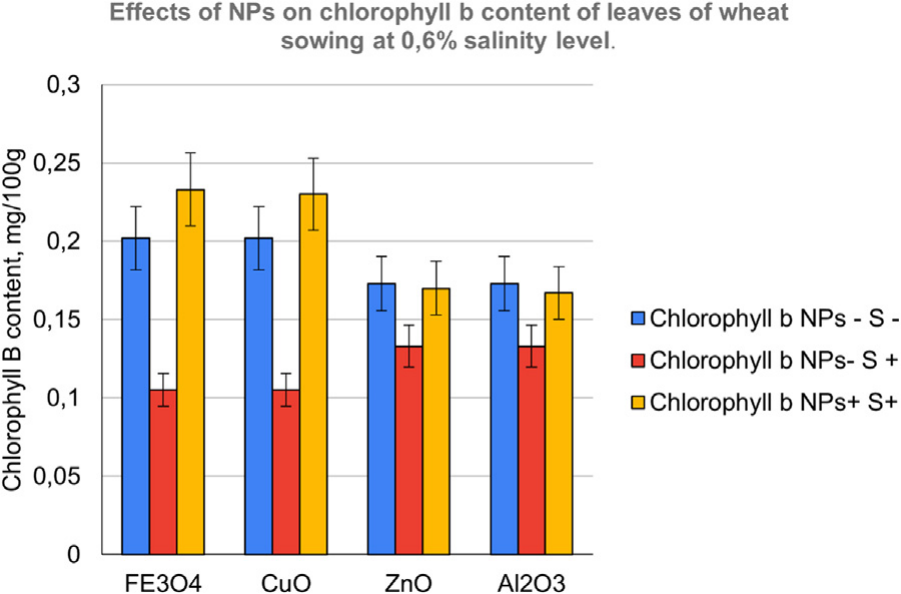
NPs- S- (not treated with nanoparticle nor salted), NPs- S+ (not treated with nanoparticle but salted), NPs+ S- (treated with nanoparticle but not salted), NPs+ S+ (treated with nanoparticle and salted).

**Fig. 9:**
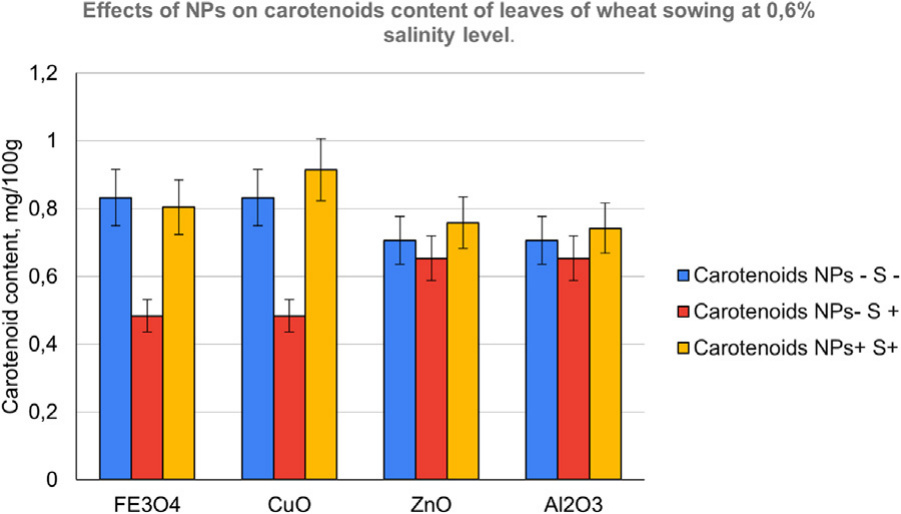
NPs- S- (not treated with nanoparticle nor salted), NPs- S+ (not treated with nanoparticle but salted), NPs+ S- (treated with nanoparticle but not salted), NPs+ S+ (treated with nanoparticle and salted).

The contents of photosynthetic pigments in the leaves of common wheat were investigated and the results are shown in Figs. 7, 8 and 9. On examining photosynthetic pigments, CuO had an overall better performance at stimulating chlorophyll a and b, and carotenoids synthesis under salt stress followed by Fe_3_O_4_ NPs. Whereas ZnO which was seen to be strongly stimulating a raise in morpho-physiological parameters exhibited poor tolerance indicating its sensitivity towards salinity followed by Al_2_O_3_

## Conclusion

This work confirms the high importance of applied sciences in agriculture and other fields in nature, and this is shown in a lot of papers published before [24], [25], [26], [27]. The results of the present study indicate that the four NPs differed in their tolerance towards salt stress. Boost in germination parameters were more pronounced in Fe_3_O_4_ NPs. Increase in shoot length was more pronounced in ZnO NPs compared to control. Increase in chlorophyll a and b, carotenoids content was more pronounced in CuO and Fe_3_O_4_ NPs compared to control. Salt has an overall negative effect on germination energy, shoot length and photosynthetic pigment content. In this experiment, seedlings on exposure to salinity showed no leaf injuries or discolouration suggesting salt stress could be due to nutritional imbalance rather than toxicity of ions. This study has also showed a contrast between NPs in salt-stressed and non-stressed wheat plants. In NaCl treated plants, NPs enhanced the germination of wheat plants while in their respective controls some NPs induced mild phytotoxicity by reducing the germination rate.It is of utmost urgency to enhance our comprehension and awareness of the gap concerning NPs, which encompasses their buildup and associated hazards. Further investigation is required to understand the fundamental mechanism.

## Funding

This work was supported by the Baku State University

## CRediT authorship contribution statement

**Adeoke Olatunbosun:** Data curation, Writing – original draft. **Huseynova Nigar:** Conceptualization, Methodology, Writing – review & editing. **Khalilov Rovshan:** Supervision. **Amrahov Nurlan:** Visualization, Investigation. **Jafarzadeh Boyukhanim:** Software. **Abdullayeva Narmina:** Software, Validation. **Azizov Ibrahim:** .

## Declaration of Competing Interest

The authors affirm that they do not have any known competing financial interests or personal relationships that could have potentially influenced the work presented in this paper.

## Data Availability

The authors do not have permission to share data.
